# New Trends in Cognitive Aging and Mild Cognitive Impairment

**DOI:** 10.3390/geriatrics7040080

**Published:** 2022-08-01

**Authors:** David Facal, Carlos Spuch, Sonia Valladares-Rodriguez

**Affiliations:** 1 Departamento de Psicoloxía Evolutiva e da Educación, Universidade de Santiago de Compostela, 15782 Santiago de Compostela, Spain; david.facal@usc.es; 2 Translational Neuroscience Group, Galicia Sur Health Research Institute (IIS Galicia Sur), SERGAS-UVIGO, Vigo, and CIBERSAM, ISCIII, 36213 Vigo, Spain; cspuch@uvigo.es; 3 Artificial Intelligence Department, Universidad Nacional de Educación a Distancia (UNED), 28040 Madrid, Spain

In this editorial, we aim to highlight some lessons learned in our field and to discuss some open questions regarding the continuum between healthy cognitive aging and dementia. Cognitive aging and cognitive impairment represent two growing realities in the current context of demographic aging, and its study requires new methodological strategies [1]. In this regard, over the last decades, longitudinal studies have gained presence and relevance in the study of cognitive aging. Life-course sociodemographic and health-related conditions can change cognitive profiles of old adults participating in these types of studies across different age cohorts. Mejía-Arango et al. (2021) [2] used data from the 2001 and 2015 years of the Mexican Health and Aging Study (MHAS) to study trends in cognitive impairment and dementia for Mexican older adults aged 60 years or older. They found a higher likelihood of dementia and a lower likelihood of cognitive impairment no dementia (CIND) in 2015 compared to 2001. Controlling for sociodemographic and health factors, these differences remain significant. Differences were found for sex, with living in rural areas and the presence of stroke being associated with increased odds of dementia (versus normal cognitive aging and CIND) in males but not in females, as well as hypertension and diabetes in females but not in males. Being aged 75 years or older was associated with increased odds of CIND (versus normal aging) in males but not in females, and being aged 75 years or older and having healthcare insurance was associated with lower odds of CIND in females but not in males. Regarding the risk of dementia, the authors interpret that those improvements in educational attainment and access to healthcare in Mexican older adults in 2015 compared to 2001 were not enough to compensate for the disadvantages of aging, rural residence, and higher levels of obesity, diabetes, hypertension, or physical disability. Regarding CIND, a higher diagnostic instability was signaled, in accordance with previous research [3,4]. None of the cardiovascular risks or their treatment showed significant effects on CIND odds. In the coming years, the improvement in the detection and prevention of degenerative processes is expected to extend life expectancy with CIND. Professionals in the sector will have to manage populations with a more uncertain cognitive status and with greater needs for stimulation and health promotion.

Another relevant trend in the study of cognitive aging in recent years is the attention paid to subjective cognitive aging. Subjective cognitive decline (SCD) has been proposed as an increased risk cognition characterized by subjective cognitive complaints that cannot be explained by other health conditions, with no evidence of objective cognitive or functional impairment [5]. Pereiro et al. (2021) [5] compared two different thresholds for complaint severity, a stricter criterion considering the 95th percentile and a less strict criterion considering the 5th percentile, showing a higher predictive validity for progression to mild cognitive impairment (MCI) and dementia using the less strict criteria. In this context of increased relevance of subjective information, different authors have started to study the accuracy of subjective reports (i.e., if self-reported or reports provided by proxies present higher predictive validity). Hatt et al. (2021) [6] studied if subjective estimations of activity participation predicted cognitive performance. Subjective estimates of activity are susceptible to different biases, including motivation, attention, emotional experiences, misunderstanding of the activities included in the questionnaires, and misunderstanding of the reported time frames. In total, 199 participants from the Activity Characteristics and Cognition (ACC) study and 170 participants from the TRAnsitions In Later Life (TRAILLs) study were studied. Both samples from different countries reported less activity participation at the weekly level than when they reported activities at a daily basis. Total activity participation did not predict cognitive status, but when activity was grouped by domains, different activity domains (e.g., cognitive, social, and emotional) significantly predicted different cognitive functions.

Cognitive status is closely linked to affective states not only in older adults without pathologies, through their own subjective status, but also in older adults with affective disorders such as depression. Caamaño et al. (2021) [7] highlight the frequent interrelation between organic pathologies, depressive symptomatology, and cognitive function in geriatric patients. The prevalence and intensity of affective disorders have increased due to the COVID-19 pandemic, although not exclusively in the geriatric population. Dosil-Díaz et al. (2021) [8] discuss the management of behavioural and psychological symptoms of dementia (i.e., BPSD) in long-term care centres, considering that the measures taken to control the spread of the virus in these centres dramatically altered the BPSD management interventions and, in general, the social and care activities with the residents. People with BPSD present higher risks of severe infection due to the relationship between frailty and dementia and their difficulties maintaining social isolation or using face masks, among others. In this context, the most relevant strategies were personalized dementia care programs and video call programs allowing social interaction between the resident and their families.

Digital technologies, including computer-based cognitive tests, are more and more common in psychogerontological and psychogeriatric practice. However, this reality is not exempt from complexities associated with its application [9]. For example, in an interesting study about the use of multimodal methods to visualize information obtained from the Sustained Attention to Response Task (SART), Rizzo et al. (2021) [10] proposes a new thresholding method based on the individual trial percentage of mistakes to determine subgroups of old adults with poor SART performance, also considering their age and Timed-Up and Go (TUG) performance. SART is a computerized continuous performance go–no go reaction time task, in which two types of mistakes can be made: commission errors (responding to no go trials) and omission errors (not responding to go trials). This novel multimodal visualization approach can help professional to better identify profiles of high risk of mobility problems in older adults. In this context of the growing use of digital technologies, immersive virtual reality (VR) can be a valuable tool to involve and motivate frail and disabled patients during therapy sessions. Orr et al. (2021) [11] studied the acceptability a series of 5 × 30 second clips of VR nature environments, showing scenes of coast and beaches with natural sounds such as breaking waves, in persons with dementia attending memory cafes. Most of the participants responded positively to the VR scenes, feeling immersed in nature with a sense of “being away” and experiencing new places. In addition, VR is considered to be able to simulate real-life situations to improve ecological validity [12]. Therefore, these technological solutions could act as effective neuropsychological tools for the assessment of everyday cognitive functions, which improve ecological validity and provide a highly enjoyable testing experience. Finally, to conclude, the use of technology in neuropsychological assessment continues to expand and enhance traditional assessment approaches. However, a number of challenges remain, such as, variations in hardware; limited information on psychometric and normative properties for different clinical populations; and the possible influence of computer literacy or other technologies on that population.

As it has been mentioned, mobility has become a central issue not only in general geriatric practice but specifically in the study of cognitive aging. Neurodegenerative diseases such as Parkinson’s disease or the functional deterioration caused by pathological aging can cause serious mobility problems in later adulthood. The ability to have proper mobility is closely linked to cognitive functioning itself, with complex networks linking the brain and the locomotor system. Rizzo et al. (2021) [10] showed that participants of the TILDA study with bad SART performances and normal TUG at baseline had higher probability to have a mobility impairment at wave 3 considering both their TUG performance and the risk of becoming a new fall. Difficulties in mobility in pathological aging can be caused neurodegenerative disease itself or vice versa, where a loss of mobility caused by a fall or lack of family care will eventually lead to cognitive impairment that, in many cases, becomes irreversible. This proposal is addressed in the article by Ries and Carroll 2021 [13], which deals with the problem of avoiding the most frequent falls through a prevention program in patients with moderate and severe dementia. They conclude that the type of format of this program determines that participation is optimal, thereby reducing the risk of deterioration caused by a fall or injury.

Complementarily, Lauretani et al. 2021 [14] propose the use of FDG-PET to measure cerebral metabolic rates of glucose in two cases of Parkinson’s patients with non-motor symptoms, so that better treatment can be achieved based on metabolic changes and not on age-independent motor deterioration. One of the great challenges facing clinical neuroscience is to detect the presence of cognitive impairment in the early stages before it becomes clinically evident. One of the ways to do this is the research and development of easily accessible molecular biomarkers. In the future, this would allow general screening of at-risk populations and, if positive, further neuroimaging and neuropsychological studies.

The articles included in this Special Issue are just a sample of the current research on cognitive aging and cognitive decline, but they allow us to address this relevant phenomenon considering the close link between cognitive status, health, and functional status in geriatric patients. When we talk about the geriatric population, we are dealing with patients with varied and different neurodegenerative diseases, comorbidities with many pathologies that can cause movement and quality of life deficits (e.g., respiratory, obesity, etc.) and polypharmacy, with a lot of medication that can alter their own quality of life. Regarding mobility, it is a matter of addressing this problem of falls in a preventive way, either through prevention programs, as proposed by Ries and Carroll [13], or by anticipating it through metabolic measures in Parkinson’s patients, as proposed by Lauretani et al. [14]. Regarding polypharmacy, it is necessary to understand the complexity of therapeutic approaches and, as highlighted by Caamaño et al. [7], the complexity of the management of these situations in different care systems. In the future, it is expected that the complexity of these situations will increase due to the pressure of population aging, for which the contribution of technologies such as the novel visualization approach proposed by Rizzo et al. (2021) [10] and the natural scenes presented with immersive VR by Orr et al. (2021) [11] will be necessary.
